# Design of a Robust System Architecture for Tracking Vehicle on Highway Based on Monocular Camera

**DOI:** 10.3390/s22093359

**Published:** 2022-04-27

**Authors:** Zhihong Wu, Fuxiang Li, Yuan Zhu, Ke Lu, Mingzhi Wu

**Affiliations:** 1 School of Automotive Studies, Tongji University, Shanghai 201804, China; zhihong.wu@tongji.edu.cn (Z.W.); yuan.zhu@tongji.edu.cn (Y.Z.); luke@tongji.edu.cn (K.L.); 2 Nanchang Automotive Institute of Intelligence & New Energy, Tongji University, Nanchang 330052, China; wumingzhi@naiine.com

**Keywords:** mono camera, multi-target tracking, Extended Kalman Filter, Navigation On Pilot, YOLO

## Abstract

Multi-Target tracking is a central aspect of modeling the environment of autonomous vehicles. A mono camera is a necessary component in the autonomous driving system. One of the biggest advantages of the mono camera is it can give out the type of vehicle and cameras are the only sensors able to interpret 2D information such as road signs or lane markings. Besides this, it has the advantage of estimating the lateral velocity of the moving object. The mono camera is now being used by companies all over the world to build autonomous vehicles. In the expressway scenario, the forward-looking camera can generate a raw picture to extract information from and finally achieve tracking multiple vehicles at the same time. A multi-object tracking system, which is composed of a convolution neural network module, depth estimation module, kinematic state estimation module, data association module, and track management module, is needed. This paper applies the YOLO detection algorithm combined with the depth estimation algorithm, Extend Kalman Filter, and Nearest Neighbor algorithm with a gating trick to build the tracking system. Finally, the tracking system is tested on the vehicle equipped with a forward mono camera, and the results show that the lateral and longitudinal position and velocity can satisfy the need for Adaptive Cruise Control (ACC), Navigation On Pilot (NOP), Auto Emergency Braking (AEB), and other applications.

## 1. Introduction

Self-driving cars or autonomous vehicles will have a huge impact on our society once the technology is deployed at scale [1]. A camera sensor has become an ingredient component in the autonomous driving system. Using this sensor, the autonomous system will be able to perform multiple tasks critical to its autonomy, such as detecting pedestrians, lanes, traffic signs, or tracking multiple moving obstacles at the same time [2]. Most important is the small package size and low manufacturing cost for the camera, allowing car manufacturers to deploy multiple cameras such as forward, backward, or side corners for environment perception.

To be safe, autonomous vehicles must be capable of perceiving the surroundings and all objects that move around. Multi-object tracking is about the accurate perception of the driving environment [3]. So, multi-target tracking based on a mono camera is a key enabling technology for any self-driving vehicle system, in which it extracts the information from the raw camera sensor to constantly estimate the state of the moving object.

The challenge of 3D visual perception mainly lies in the following facts: (1) image is the projection of the real-world object; in the image plane in the projection transformation the distance information would be lost; (2) the size of the object on the image would change according to the distance; (3) it is hard to estimate the object’s size and distance. To solve the above challenges, some solutions are proposed such as (1) by integrating another kind of sensor, for example, Lidar; (2) by applying some geometry constraints; (3) by deploying a deep learning method to extract 3D information from an image; or (4) by using multiple cameras or stereo algorithm. Regarding 3D mono camera perception, the related work can be categorized into four types: (1) based on inverse transformation from 2D to 3D, (2) based on key points and 3D model, (3) based on the constraints between 2D and 3D, and (4) directly extract 3D information through deep learning.

In category 1, the typical method is BEV-IPM [4], which transforms the 2D image into the BEV image with the assumption that the ground plane and vehicle coordinate are parallel with the Cartesian coordinates, and then feeds the BEV map into the YOLO network to detect the bottom line of the object. However, it is hard to guarantee the flat of the road. Another representative method is Pseudo-Lidar [5], which transforms the image to 3D point cloud data based on the depth image and fuses point cloud and image to detect the 3D object. The core of this algorithm is depth estimation.

In category 2, the classical method is DeepMANTA [6], which obtains the 2D bounding box through a deep neural network, 2D keypoints set, and the keypoints visibility and the similarity with the 3D model. After combining the 3D model with the 2D keypoints, the algorithm can output the 3D information. The disadvantage of this method is that it needs a 3D model of the object; the 3D model is limited as, in the highway scenario, there are many types of cars.

In category 3, the typical method is Deep3DBox [7], which outputs the 3D information with the constraint condition that it can be found at least one corner of the 3D bounding box at the 2D bounding box edge. This constraint condition is modeled as a network layer. So, this method can be trained from end to end. In a real scene, it is hard to meet the geometry assumption. In category 4, there exist two types: anchor-based and anchor-free. In an anchor-based method, it produces a dense candidate 3D bounding box according to the prior knowledge and then projects the 3D bounding box to the 2D image. After scoring the 2D candidate bounding box, it outputs the final candidates. The typical method is Mono3D [8], which produces the 3D candidate bounding box based on the prior position and size of the object. After projecting the 3D bounding box to the 2D image, it grades the 2D bounding box according to the feature from segmentation, size, and position and finally gives out the proposal. The biggest advantage of this method is that the dense anchor would burden the computation. Another classical method is TLNet [9], in which the size and orientation of the anchor are defined. It applies the detection from 2D images forming the frustum to decrease the number of anchors. In the anchor-free method, the 3D information is regressed directly from the image. The representative method is FCOS3D [10], which is similar to the 2D object detection with the addition of the 3D object regression head. The car 3D average precision of FCOS3D is about 11.8% with the IoU more than 0.7 on the KITTI bench, indicating that it is far from practical application.

The complexity of the system lies in the fact there are no perfect mono camera detectors and this means that it is susceptible to two kinds of errors: missed detections and false detection. Besides this, under the highway scenario, the number of moving objects in the field of view is unknown. It is difficult to know the state of the moving objects or where they are located and where they are going. Moreover, the multi-target tracking system is often restricted to tracking the objects that are inside the field of view of the mono camera [11], and the kinematic objects may appear or disappear from the field of view. To overcome these challenges and at the same time keep the advantage of the mono camera, a multi-object tracking system based on the mono camera is designed in this paper. It is composed of three main modules: the object detector [12], the depth estimator [13], and the multi-target tracking [3] engine.

The object detector module uses a deep learning approach to detect vehicles in mono camera images, which can obtain a set of bounding boxes around all vehicles in the scene. In the past, methods for object detection were often based on histograms of oriented gradient (HOG) [14] and support vector machine (SVM) [15] until the advent of deep learning or convolutional neural networks. Deep learning algorithms are now the state of the art in most computer vision problems, such as object detection in every self-driving system such as two stages objector RCNN family [16] and one stage detector YOLO [17] series or SSD [18]. However, using deep learning adds additional constraints to the system because it requires more computational power. In an autonomous vehicle, one of the major concerns of the deep learning object detector is the speed. In contrast to other methods, the major advantage of the YOLO is its speed and it can be deployed in the autonomous system easily, so this paper adopts YOLO as the object detector of a multi-object tracking system.

In the mono camera system, every 3D object in the world is projected through the lens onto the image plane. The shortcoming of a monocular camera is that it will lose the depth information and cannot directly resolve this ambiguity; this paper resolves it through the depth estimator.

In the mono camera-based multi-target tracking system, another problem to deal with is the data association, which determines which measurement comes from which measurement. The Probabilistic Data Association (PDA) series methods are adopted in the literature [19]. These methods share a similar procedure of data association, in that they first compute the probabilities of being correct for each validated measurement at the current time and then weight these probabilities to obtain the state estimate of the target. Another method is the Hungarian method [20]. The Hungarian Algorithm solves the track and measurement assignment problem with the runtime complexity worst-case $O\left(n^{3}\right)$. This paper adopts the global nearest neighbor with the gating trick, which is not only reduces the data association complexity but is also implemented easily.

In the algorithm of target motion state estimation, from the perspective of the highway driving scenario, this paper applies a linear Constant Acceleration (CA) motion model and non-linear observation model, since the camera measures the object in the image coordinates and needs to convert it to the ego-vehicle Cartesian coordinates. Extended Kalman Filter (EKF) [21] is widely used in nonlinear filtering, in which exist some nonlinear factors.

Track management is another crucial problem in the multi-target tracking system, which refers to the track initialization, maintenance, and cancellations because the moving objects may enter or disappear from the mono camera sensor field of view [3].

**Figure 1:**
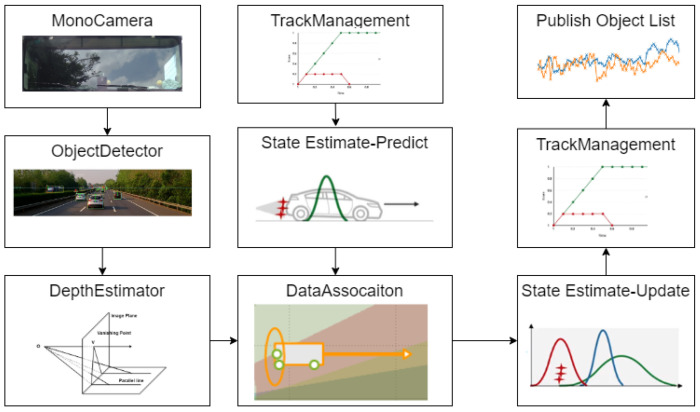
Multi-target tracking system based on mono camera.

Due to the analysis of the above module, the mono camera-based multi-target tracking framework proposed in this paper is shown in Figure 1.

The rest of the paper is organized as follows. Section 2 discusses the deep learning-based object detector YOLO and the depth estimator based on the bounding boxes published by the object detector. Section 3 talks about the kinematic transition model of the moving object and the mono camera sensor measurement model. Section 4 analyzes how to apply the nonlinear filter approach Extended Kalman Filter in the mono camera tracking system. In Section 5, a gating method combined with the data association method Hungarian is proposed. In Section 6, this work adopts a simple track management policy. Finally, the performance of the mono camera tracking system is evaluated qualitatively and quantitatively.

The contribution of this paper can be summarized as follows:

1. A multi-target tracking system based on a mono camera is constructed, which can be used on the expressway scene
2. An object detector combined with a depth estimator is designed to resolve the mono camera depth lost problem.
3. The whole system is tested under the highway scenario and the performance of the lateral and longitudinal is evaluated qualitatively and quantitatively.

## 2. The Object Detector and Depth Estimator

The object detector module adopts the deep learning approach YOLO. YOLO uses a single neural network for the full image. The network divides the image into regions and predicts bounding boxes and probabilities for each region. One of the major advantages of the YOLO framework is its speed. In contrast to other methods, it only makes a single pass through the neural network. When compared with its peers, such as SSD or RCNN family, the results are quite decent.

**Figure 2:**
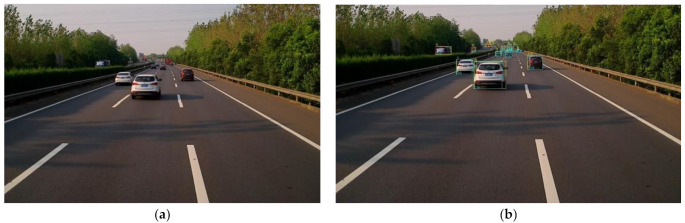
Comparison between the raw mono camera image and the result after the object detector. (**a**) is the raw image; (**b**) is the detected vehicle.

In the YOLO algorithm, the mono camera image is divided into a 13 × 13 grid of cells. Based on the size of the input image, the size of these cells in pixels varies. Each cell is then used for predicting a set of bounding boxes. For each bounding box, the network also predicts the confidence that the bounding box encloses a particular object as well as the probability of the object belonging to a particular class. Lastly, non-maximum suppression is used to eliminate bounding boxes with a low confidence level as well as redundant bounding boxes enclosing the same object. The result after feeding the mono camera image to the object detector can be seen in Figure 2.

**Figure 3:**
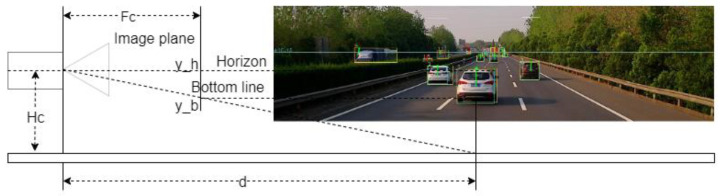
The depth estimator.

In the mono camera system, every 3D object in the real world is projected to the 2D image plane, which loses the depth information. Thus, how to recover the depth information in the mono camera image is critical for multiple vehicle tracking. The depth estimator is shown in Figure 3.

As shown in Figure 3, the distance can be computed by the following Equation (1):

(1) $$\frac{F_{c}}{d}=\frac{y_{b}-y_{h}}{H_{c}}$$

where, $H_{c}$ is the height mounted on the ego vehicle, $F_{c}$ is the focal length of the mono camera, $y_{h}$ is the pixel location of the vanishing point, and $y_{b}$ is the pixel location of the bottom line in the image plane.

## 3. System Model

**Figure 4:**
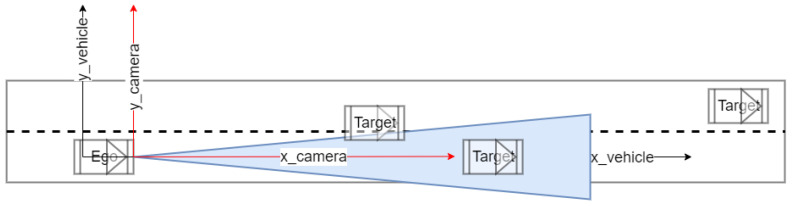
Mono camera-based multi-target tracking system.

After obtaining the detections from the object detector and depth estimator, there exists the bounding boxes information, the moving vehicle type information, and the distance to the ego vehicle. So, the mono camera tracking system is about to study the estimation of time-varying parameters, that is, the state estimation problem which refers to smoothing the past motion state of a target, filtering the present motion state, and predicting the future motion state of a target [17]. A typical forward-looking mono camera multi-target tracking system can be seen in Figure 4.

In the highway scenario, this paper adopts the constant velocity motion model as the system state transition model. A system model of an object is represented by a Cartesian position and velocity components. The model assumes the motion of target vehicle with constant velocity in lateral and longitudinal direction and implements noise for the velocity components using two independent Wiener processes. The position, velocity, and acceleration can be expressed in the form as Equations (2)–(5):

(2) $$x_{k+1}=x_{k}+{\dot{x}}_{k}T+\frac{1}{2}{\ddot{x}}_{k}T^{2}+\frac{1}{2}\nu_{x}T^{2}$$

(3) $$y_{k+1}=y_{k}+{\dot{y}}_{k}T+\frac{1}{2}{\ddot{y}}_{k}T^{2}+\frac{1}{2}\nu_{y}T^{2}$$

(4) $${\dot{x}}_{k+1}={\dot{x}}_{k}+{\ddot{x}}_{k}T+\nu_{x}T$$

(5) $${\dot{y}}_{k+1}={\dot{y}}_{k}+{\ddot{y}}_{k}T+\nu_{y}T$$

Assuming that the state space is $X\left(k\right)={\left[x_{k+1}\;y_{k+1}\;{\dot{x}}_{k+1}\;{\dot{y}}_{k+1}\right]}^{T}$ and the process noise vector is $\nu \left(k\right)={\left[\nu_{x}\;\nu_{y}\;\right]}^{T}$ The corresponding state transition matrix and process matrix are respectively:

(6) $$A\left(k\right)=\left[\begin{array}{cccc} 1 & 0 & T & 0\\ 0 & 1 & 0 & T\\ 0 & 0 & 1 & 0\\ 0 & 0 & 0 & 1 \end{array}\right]\;B\left(k\right)=\left[\begin{array}{cc} 0.5T^{2} & 0\\ 0 & 0.5T^{2}\\ T & 0\\ 0 & T \end{array}\right]$$

**Figure 5:**
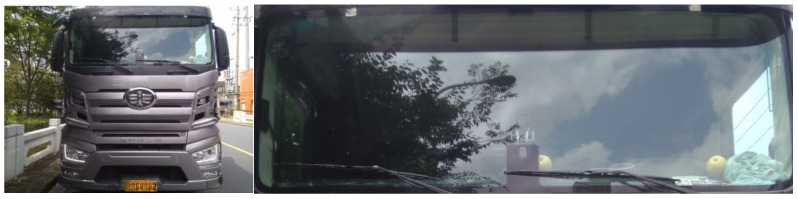
The front-facing mono camera.

The front-facing mono camera is mounted on the wind window of the vehicle as shown in Figure 5.

**Figure 6:**
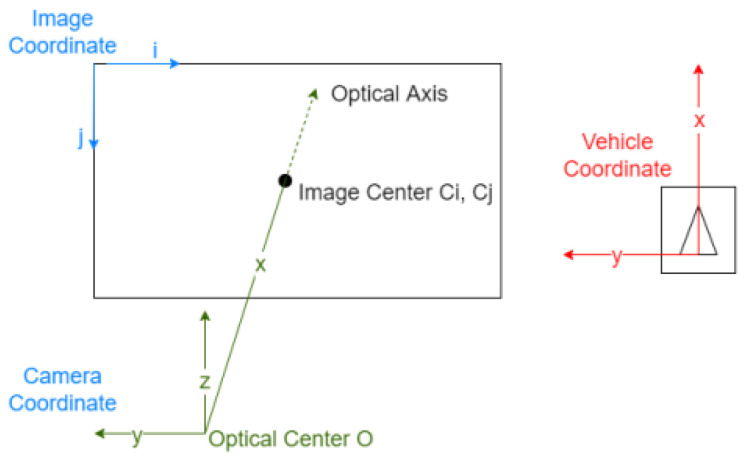
The coordinates in the mono camera-based tracking system.

In the mono camera measurement system, the vehicles in the real world are projected to the image plane. In a multi-object tracking system, it uses an ego-vehicle coordinate system for tracking the moving objects where there exist three types of coordinates, namely: the ego-vehicle coordinate system, the mono camera coordinate system, and the Image coordinate system, as shown in Figure 6.

As shown in Figure 6, in the camera coordinate system, the *x*-axis is the camera’s optical axis. The intersection of the optical axis and the image plane is called the image center or principle point. In the vehicle coordinate system for tracking, the *x*-axis points forward, the *y*-axis points to the left, and the *z*-axis points upward. The image coordinate system usually has its origin in the upper left corner of the image. The pixel coordinates are denoted by i for the horizontal dimension and j for the vertical dimension. Note that the pixel is not necessarily perfectly square. Instead of a single focal length f, it may have two numbers $\left(f_{i}\;f_{j}\right)$ that might slightly differ. The image center C and the focal length f are derived through intrinsic camera calibration.

The mono camera measurement model can be defined as Equation (7):

(7) $$h\left(X\right)=h\left(\left(\begin{array}{c} p_{x}\\ p_{y}\\ v_{x}\\ v_{y} \end{array}\right)\right)=\left(\begin{array}{c} c_{i}-\frac{f_{i}p_{y}}{p_{x}}\\ c_{j}-\frac{f_{j}p_{z}}{p_{x}} \end{array}\right)$$

In Equation (6),$\;\left(p_{x}\;p_{y}\;p_{z}\right)$ is the 3D position of the vehicle in the real world. The vehicle is projected to the image plane. This is the mono camera measurement model $h\left(X\right)$, the formula summarizes how to compute the image coordinates from a 3D object in vehicle coordinate. Projecting a 3D point to a 2D image plane space makes Equation (6) a nonlinear measurement function. Hence, for a mono camera, it needs to calculate the mapping to convert from Cartesian coordinates to image coordinates. So, the mono camera measurement equation can be defined as Equation (8):

(8) $$z=h\left(X\right)+\omega$$

In Equation (7), z is the measurement vector, and ω is a white Gaussian measurement noise sequence with zero mean and covariance. As can be seen from Equation (7), the measurement function is nonlinear; in the next section, this paper will talk about how to deal with the nonlinear with the Extended Kalman Filter.

## 4. The State Estimator

The most famous state estimator is the Kalman filter [18], which obtains dynamic estimation of the moving targets under the linear Gaussian assumption, but in many actual cases, the measurement function is non-linear, as shown in Equation (8). The usual approach to turning nonlinear filtering into approximate linear filtering is by using linearization techniques and then applying linear filtering theory to the suboptimal filtering algorithm for the original nonlinear filtering problems. The most commonly used linearization method is the Taylor series expansion, by which the filtering method of the Extended Kalman Filter is achieved [19].

The mono camera measurement function $h\left(X\right)$ is composed of two equations that show how the predicted state is mapped into the measurement space, as shown in Equation (6). After calculating all the partial derivatives, our resulting Jacobian matrix $H_{j}$ is defined as Equation (8):

(9) $$H_{j}=\left(\begin{array}{cccc} \frac{\partial h_{1}\left(X\right)}{\partial p_{x}} & \frac{\partial h_{1}\left(X\right)}{\partial p_{y}} & \frac{\partial h_{1}\left(X\right)}{\partial v_{x}} & \frac{\partial h_{1}\left(X\right)}{\partial v_{y}}\\ \frac{\partial h_{2}\left(X\right)}{\partial p_{x}} & \frac{\partial h_{2}\left(X\right)}{\partial p_{y}} & \frac{\partial h_{2}\left(X\right)}{\partial v_{x}} & \frac{\partial h_{2}\left(X\right)}{\partial v_{y}} \end{array}\right)=\left(\begin{array}{cccc} \frac{f_{i}p_{y}}{p_{x}^{2}} & -\frac{f_{i}}{p_{x}} & 0 & 0\\ \frac{f_{j}p_{z}}{p_{x}^{2}} & 0 & 0 & 0 \end{array}\right)$$

So, after linearizing the measurement equation, the transition and measurement equation are both linear equations. So, it can use the Standard Kalman Filter to predict and update the track state in the mono camera-based tracking system. The Kalman Filter includes two steps: prediction and update, and the process is shown as Equations (10)–(16):

The prediction step is defined as Equation (10):

(10) $$\hat{x}\left(k+1|k\right)=A\left(k\right)\hat{x}\left(k|k\right)$$

The state prediction covariance is Equation (11):

(11) $$p\left(k+1|k\right)=A\left(k\right)p\left(k+1|k\right)A{\left(k\right)}^{T}+Q\left(k\right)$$

The updated state estimate is shown as Equation (12):

(12) $$\hat{x}\left(k+1|k+1\right)=\hat{x}\left(k+1|k\right)+W\left(k+1\right)\nu \left(k+1\right)$$

$W\left(k+1\right)$ is the filter gain defined as Equation (13):

(13) $$W\left(k+1\right)=p\left(k+1|k\right)H{\left(k+1\right)}^{\prime }S{\left(k+1\right)}^{-1}$$

$\nu \left(k+1\right)$ is called the innovation or measurement residual defined as Equation (14):

(14) $$\nu \left(k+1\right)=z\left(k+1\right)-\hat{z}\left(k+1|k\right)$$

$S\left(k+1\right)$ is the measurement residual covariance following Equation (15):

(15) $$S\left(k+1\right)=H\left(k+1\right)p\left(k+1|k\right)H{\left(k+1\right)}^{T}+R\left(k+1\right)$$

Finally, the updated covariance of the state at time k+1 follows Equation (16):

(16) $$p\left(k+1|k+1\right)=p\left(k+1|k\right)-W\left(k+1\right)S\left(k+1\right)W{\left(k+1\right)}^{T}$$

In the mono camera multi-target tracking system, by iterating between the prediction and update steps, it can maintain the states of the tracked objects. This mechanism can be tuned by specifying if the system should rely more on the motion model assumption or the measurement by specifying noise parameters for both. Measurement noise is typically specified by the sensor manufacturer and is based on the physical characteristic of the sensor as to how accurate it is. Process noise is the parameter that accounts for unknown or unmodeled motion. The ratio of process noise to measurement noise determines whether the tracking system relies more on process versus measurements.

## 5. Data Association

Data association is about what is being associated with what. On highway driving scenario, data association decides which track to update with which measurement. The data association module calculates track and measurement pairs and tells which measurement probably originated from which track. For the association, two assumptions are made: each track generates at most one measurement and each measurement originates from at most one track. A simple approach is to update the track with the closet measurement. This paper uses the Mahalanobis distance as the metric for decision, the Mahalanobis distance is defined as Equation (17), where z is the measurement, x is the position, and S is innovation covariance.

(17) $$d\left(x,z\right)={\left(z-h\left(x\right)\right)}^{T}S^{-1}\left(z-h\left(x\right)\right)$$

**Figure 7:**
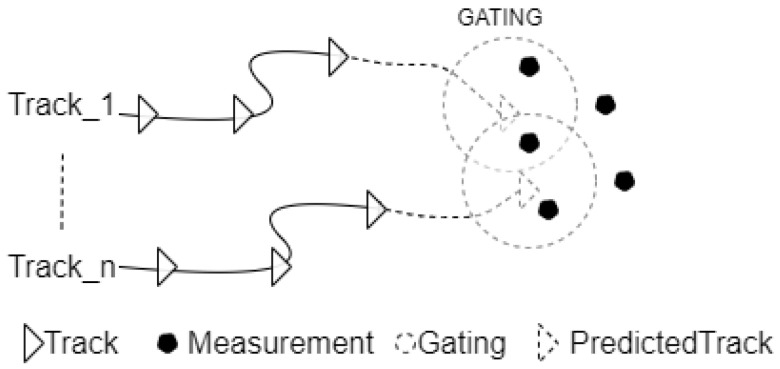
The Gating Mechanism.

To decrease the computational effort to calculate all possible distances, it does not make sense to calculate the distances of very unlikely and faraway combinations. By defining a gate or threshold to the Mahalanobis distance, for every possible association between a track and a measurement, it must be first checked whether the Mahalanobis distance is smaller than the threshold; if the distance is bigger, ignore this possible association. The gating trick is shown in Figure 7.

If the measurement lies outside a track’s gate, the distance in the data association matrix is set to infinity as shown by Equation (18):

(18) $$A=\left(\begin{array}{cccc} d\left(1,1\right) & \propto & \ldots & d\left(1,m\right)\\ . & . & . & .\\ . & . & . & .\\ . & . & . & .\\ d\left(n,1\right) & \propto & \ldots & d\left(n,m\right) \end{array}\right)$$

In data association, it is assumed that each track generates at most one measurement and each measurement originates from at most one track. Suppose there are N tracks and M measurements. The association matrix A is NxM matrix that contains the Mahalanobis distance between each track and each measurement.

There also need a list of unassigned tracks and a list of unassigned measurements. This paper looks for the smallest entry in A to determine which track to update with which measurement, then delete this row and column from A and the track ID and measurement ID from the lists, and repeat this process until A is empty.

**Figure 8:**
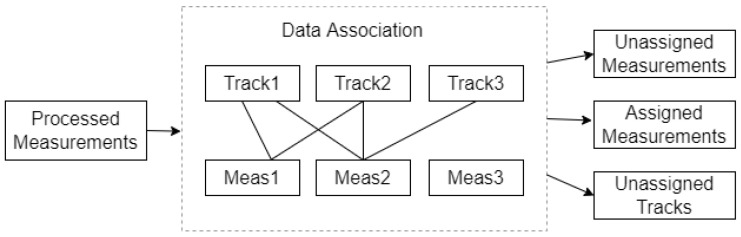
Data Association Results.

When the data association module is updated with new set of detections from the mono camera, the tracker attempts to assign these detections to the existing tracks it maintains. The assignment has three possible outcomes: detection is left unassigned, detection is assigned to a track, and a track is left unassigned as depicted in Figure 8. If the assignment gating or threshold is small, there is a chance that much detection is left unassigned, leading to the creation of many tracks. If it is too large, then an incorrect detection association may happen.

## 6. Track Management Strategies

**Figure 9:**
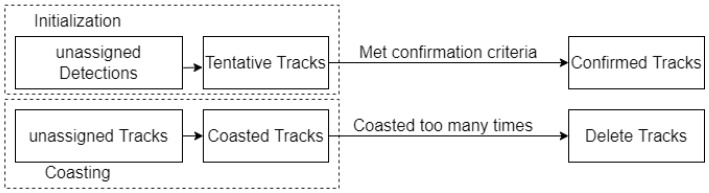
Track Management Strategies.

A track has a lifecycle of initialization, confirmation, update, coasting, and deletion. The mono camera-based multi-target tracking system must have its track management module. The track management strategies adopted by this paper are shown in Figure 9.

A track is initialized from an unassigned detection. If the detection is not classified as anything, the track is initialized as tentative, which means that the tracker is uncertain whether the track is a false alarm or a real object. The track is confirmed when a classified detection is assigned to it or it meets the confirmation criteria set by the confirmation threshold. This is a 2-element vector [M,N], which means that tentative tracks will be confirmed if assigned at least M detections are made in a span of N time steps. If the track is left unassigned, it is coasted. The tracker then has to decide to either remove these tracks or keep them in the chance that it might be updated shortly. This coasting period is controlled by the deletion threshold. If the tracker goes above these numbers of updates and an existing track is still not updated, then that track is deleted. In this paper, 6 means that a track will be deleted if it does not receive any assignments six times steps in a row.

To test and evaluate the dynamic performance of the mono-based tracking system in the following part this paper designs two validation platforms one is based on the Lidar sensor and the other is based on RTK. The Lidar sensor is another type of sensor in autonomous vehicles and has the advantage of measuring position, but the price is much higher compared with a mono camera. So, this paper makes a comparison between the camera and Lidar. Besides this, to improve tracking accuracy another experiment is done based on RTK.

## 7. Performance Evaluation

### 7.1. Construction of the Performance Analysis Platform

**Figure 10:**
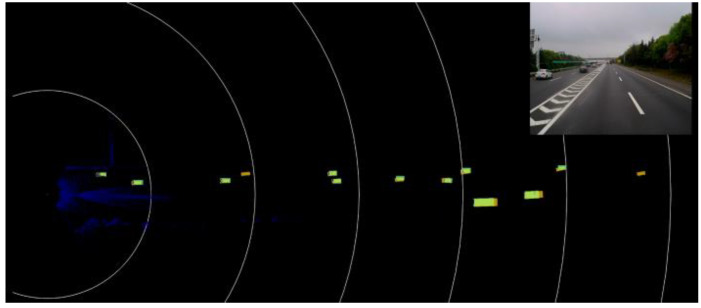
The Lidar ground Truth.

The test and validation platform is composed of front-view Lidar and a front-facing mono camera, as depicted in Figure 10, in which the perception result of the Lidar is the *baseline* to evaluate the performance of the mono camera-based multi-object tracking system.

### 7.2. Evaluation of Vehicle Detector

**Figure 11:**
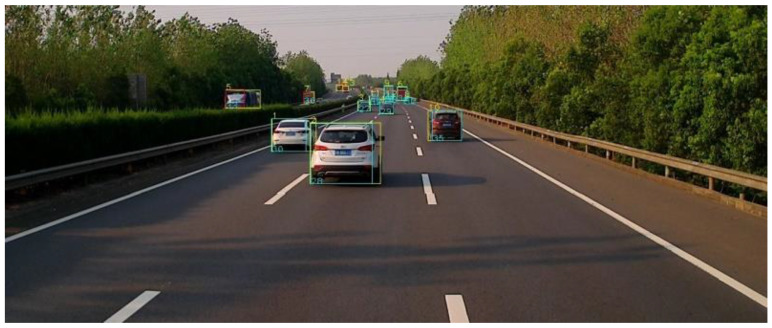
The result of the vehicle detector.

The test and validation of the YOLO-based detector is done under the highway scenario with good weather condition as show in Figure 11. This paper divides the objects on the highway into three classes: vehicle, car and truck. The whole scene is composed of a total of 16,859 objects and the detector detects 15,730 objects successfully. The performance of the detector is shown in Table 1.

As shown in Table 1, the average precision (AP) for all classes is more than 85% but less than 65% for truck. At the precise and recall index, the detector performance at vehicle and car is better than at truck. The recall of the truck is less than 68%. As truck is very common under highway scene and this may have potential hazards. In the future this paper will focus on improving recall and precision of the truck by adding more truck data into the training data set.

### 7.3. Evaluation of Experimental Data

**Figure 12:**
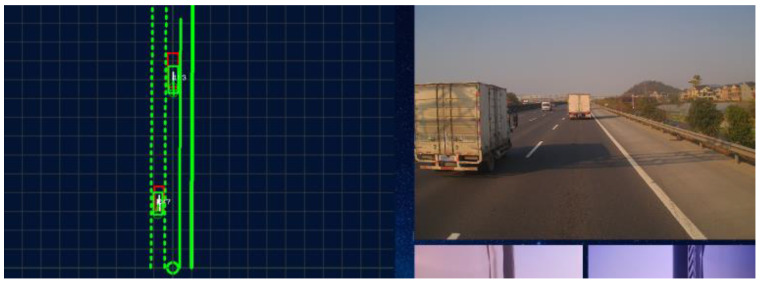
Mono camera-based tracking system.

As shown in Figure 12, which is on the highway test scene, there are two trucks: one is in the front of the ego vehicle and the other is in the left lane of the ego vehicle. The red rectangle with the truck is the tracking result of the Lidar and the green rectangle with the truck is the tracking result of the mono camera.

**Figure 13:**
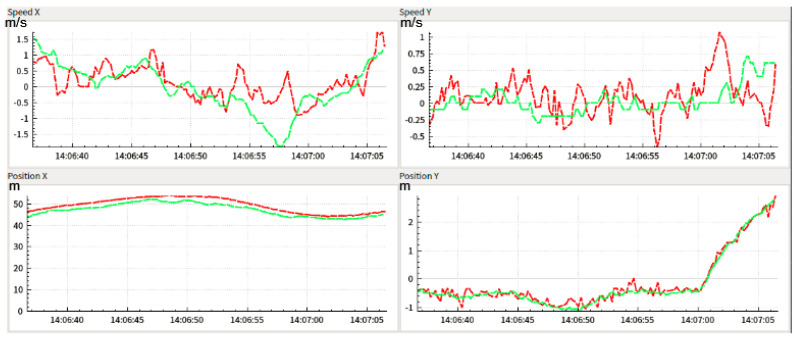
The longitudinal and lateral velocity and position of the target vehicle relative to the ego-vehicle.

To analyze, the performance of the proposed algorithm this paper adopts the Lidar perception result as the baseline. In Figure 13, the red curve line is the result of the Lidar and the green curve line represents the mono camera perception output. The max longitudinal position error is no more than 5 m. The lateral position error is no more than 0.5 m.

## 8. Further Validation

### Validation Platform

**Figure 14:**
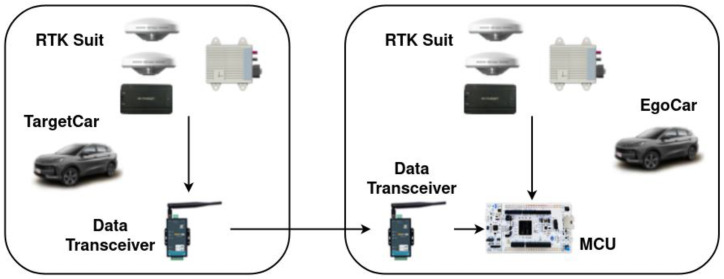
Validation platform.

The validation platform is composed of the ego-car and target car that are both quipped with the RTK suit and data transceiver suit as shown in Figure 14.

**Figure 15:**
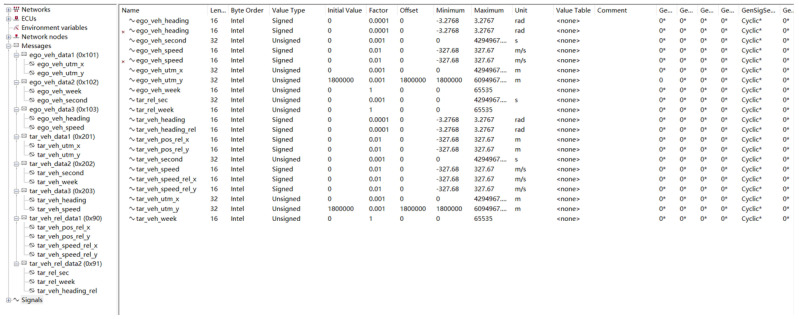
The CAN signal.

The target car transfers the kinematic information to the ego car through the data transceiver and finally reaches the MCU board. The MCU board also receives the information from the ego car through series communication and then processes the data from the ego car and target car, then obtains the kinematic information of the target car in the ego car coordinate system. Finally, the result is sent out through the CAN bus. The CAN signal is shown in Figure 15.

**Figure 16:**
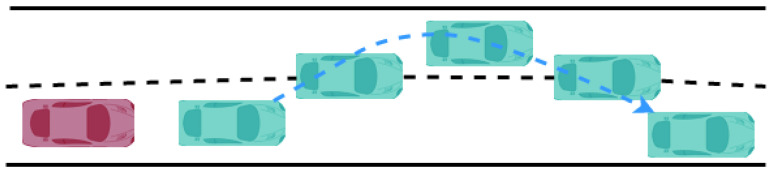
Test Scene.

The test scenario is shown in Figure 16. The ego car drives with constant velocity and the target car moves in the front of the ego car with the action acceleration, deceleration, cut in, and cut out.

The compared state includes the position and velocity in both lateral and longitudinal direction as expressed by the following Equations (19)–(22):

(19) pos_x_diff= pos_x_camera−pos_x_rtk

(20) pos_y_diff= pos_y_camera−pos_y_rtk

(21) vel_x_diff= vel_x_camera−vel_x_rtk

(22) vel_y_diff= vel_y_camera−vel_y_rtk

where,

pos_x_diff is the longitudinal position difference between mono camera and RTK;
pos_y_diff is the lateral position difference between mono camera and RTK;
vel_x_diff is the longitudinal velocity difference between mono camera and RTK;
vel_y_diff is the lateral velocity difference between mono camera and RTK.

**Figure 17:**
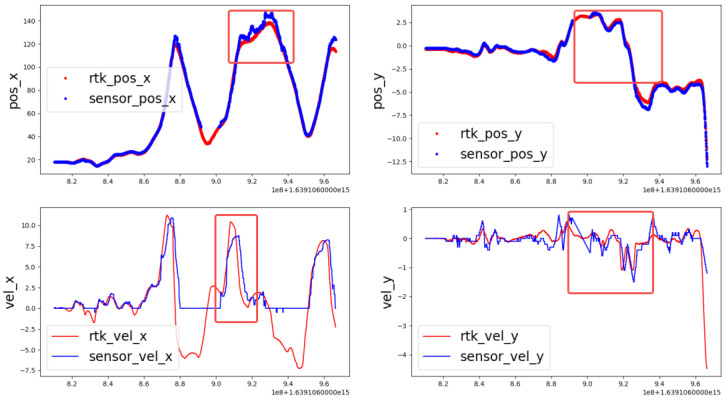
Tracking result between mono camera and RTK.

**Figure 18:**
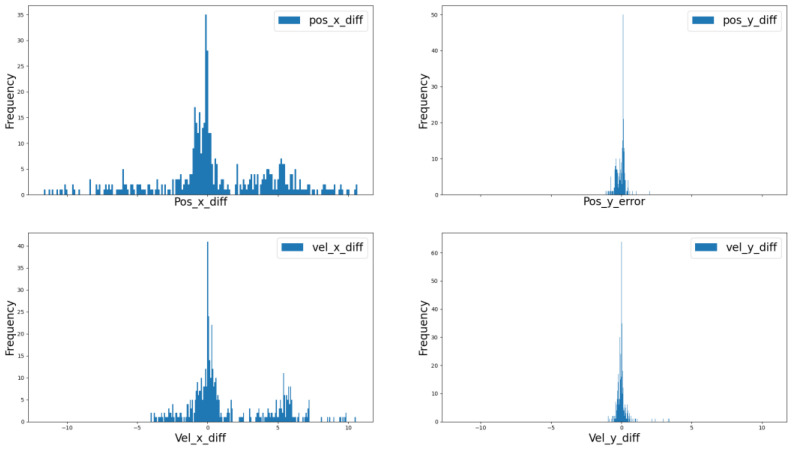
Error frequency distribution.

From Figure 17, it can be seen that the mono camera-based tracking system has better performance in lateral compared with longitudinal. From Table 2, it can be seen the position accuracy in longitudinal is less than 1.13 m and in the lateral is less than 0.11 m, which indicates that the mono camera system has better lateral accuracy. However, there exists disturbance in both the lateral and longitudinal velocity. Besides this, when the target car has maneuvers such as acceleration, deceleration, cut in, or cut out, the performance will decline as shown in the red frame. The longitudinal and lateral position and velocity error frequency can be seen in Figure 18.

For the autonomous system to have a comprehensive and accurate understanding of the surrounding dynamic environment, it is necessary to integrate more types of sensors to compensate for each other. In the future, we will integrate the Radar sensor into the tracking system to improve its performance of the tracking system. Additionally, the robustness of the tracking system under different light and weather conditions is another big problem needed to address in future work.

## 9. Conclusions

This paper designs a robust tracking system based on a mono camera. According to the characteristics of the mono camera, a YOLO-based detector is used to give out the bounding boxes and a vanishing point-based depth estimator is used to estimate the distance to compensate for the lost depth information caused by projecting from the 3D Cartesian coordinate to the 2D image plane. To track the vehicle on the highway, a constant velocity model is used in this paper and the Extended Kalman filter is applied to deal with the mono camera nonlinear measurement problem. Nearest neighbor with a gating trick is adopted to handle the data association problem. Besides these, a track management strategy is proposed to initialize, maintain, and delete tracks. Finally, to evaluate the mono tracking system, a Lidar-based ground truth method is proposed. The research results in this paper provide a good and beneficial reference for the autonomous driving system based on a mono camera.

## Figures and Tables

**Table 1:** The performance of the detector at different class.

	All_Class	Vehicle	Car	Truck
AP	0.889	0.894	0.865	0.641
Precise	0.878	0.870	0.829	0.777
Recall	0.933	0.939	0.925	0.679

**Table 2:** The statistics under the test scene.

	Mean	Variance	95% Distribution
pos_x_error	1.123	7.355	7.091
pos_y_error	−0.104	0.068	0.187
vel_x_error	1.352	6.782	6.230
vel_y_error	0.008	0.155	0.440

## Data Availability

Not applicable.
